# Lack of Ecto-5′-Nucleotidase Protects Sensitized Mice against Allergen Challenge

**DOI:** 10.3390/biom12050697

**Published:** 2022-05-13

**Authors:** Elisabetta Caiazzo, Ida Cerqua, Roberta Turiello, Maria Antonietta Riemma, Giacomo De Palma, Armando Ialenti, Fiorentina Roviezzo, Silvana Morello, Carla Cicala

**Affiliations:** 1 Department of Pharmacy, School of Medicine and Surgery, University of Naples Federico II, 80131 Naples, Italy; elisabetta.caiazzo@unina.it (E.C.); ida.cerqua@unina.it (I.C.); mariaantonietta.riemma@unicampania.it (M.A.R.); giacomo.depalma@unina.it (G.D.P.); armando.ialenti@unina.it (A.I.); fiorentina.roviezzo@unina.it (F.R.); 2 Department of Pharmacy, University of Salerno, 84084 Fisciano, Italy; rturiello@unisa.it (R.T.); smorello@unisa.it (S.M.); 3 PhD Program in Drug Discovery and Development, University of Salerno, 84084 Fisciano, Italy

**Keywords:** airways, allergy, CD73, ecto-5′-nucleotidase, mast cells, hyperresponsiveness, B cells, CD23, sensitization

## Abstract

Ecto-5′-nucleotidase (CD73), the ectoenzyme that together with CD39 is responsible for extracellular ATP hydrolysis and adenosine accumulation, regulates immune/inflammatory processes by controlling innate and acquired immunity cell functions. We previously demonstrated that CD73 is required for the assessment of a controlled allergic sensitization, in mice. Here, we evaluated the response to aerosolized allergen of female-sensitized mice lacking CD73 in comparison with their wild type counterpart. Results obtained show, in mice lacking CD73, the absence of airway hyperreactivity in response to an allergen challenge, paralleled by reduced airway CD23^+^B cells and IL4^+^T cells pulmonary accumulation together with reduced mast cells accumulation and degranulation. Our findings indicate CD73 as a potential therapeutic target for allergic asthma.

## 1. Introduction

Sensitization to an allergen is the condition for an allergic reaction to occur. Usually, humans recognize that they are sensitized to an allergen only when they come in contact with the allergen and experience the allergic reaction, which can even be fatal. Innate and adaptive immune systems talk to each other to assess allergic sensitization [1]. Sensitized subjects undergo different allergic manifestations when they encounter the allergen, depending on individual, genetic, and environmental factors [2].

Exploring host factors controlling the assessment of sensitization and those that control effector mechanisms of allergy is needed to identify potential therapeutic targets. Ecto-5′-nucleotidase (CD73) is the ectoenzyme responsible for tissue extracellular adenosine accumulation [3]. There is evidence that extracellular adenosine accumulates in asthmatic airways and plays a role in bronchial inflammation and hyperreactivity. An association between exposure to an allergen and airway responsiveness to adenosine and AMP has been demonstrated in experimental animals and humans [4,5,6,7,8]. CD73 regulates immune/inflammatory processes by controlling innate and acquired immunity cell functions [9,10,11,12,13]. By using a model of ovalbumin (OVA)-sensitized female mice, we previously demonstrated that CD73 is required for the assessment of a controlled sensitization. Indeed, sensitized mice lacking CD73 develop exacerbated airway inflammation, characterized by increased levels of Th2 cytokines and reduced frequency of regulatory T cells into the lung; nonetheless, they did not develop airway hyperreactivity [14]. The aim of the present work was to investigate the role of CD73 in the effector phase induced by an allergen challenge in the model of sensitized mice. We evaluated the response to aerosolized allergen of female-sensitized mice lacking CD73. Results obtained show, in mice lacking CD73, the absence of airway hyperreactivity in response to an allergen challenge, paralleled by reduced airway CD23^+^B cells and IL4^+^T cell pulmonary accumulation together with reduced mast cell accumulation and degranulation. Although further in-depth analysis is required, our findings suggest that targeting CD73 might be a therapeutic strategy to control allergic airway hyperresponsiveness. 

## 2. Materials and Methods

### 2.1. Animals

Female C57BL/6J mice (Charles River, Calco, Italy) and CD73^−/−^ were bred and housed in the animal facilities laboratory of Department of Pharmacy at the University of Naples Federico II. All animal procedures were approved by the Italian Ministry of Health and were in accordance with Italian (DL 26/2014) and European (n.63/2010/UE) guidelines and regulations.

### 2.2. Allergic Sensitization and Challenge

Mice (8 weeks old) were sensitized with 100 µg of ovalbumin (OVA, grade V; Sigma Aldrich, Milan, Italy) emulsified with aluminium hydroxide (Al(OH)_3_, 13 mg/mL) and administered subcutaneously (s.c) on days 1 and 8. From day 21, mice were challenged with aerosolized OVA (5% *w*/*v* in saline) or with saline for 2 consecutive days for 20 min each day. All mice were sacrificed in 24 h following the last OVA or saline challenge.

### 2.3. Immunoblot Analysis for CD73

Pulmonary tissue was homogenized using a FastPrep-24 instrument (MP Biomedicals, Santa Ana, CA, USA) as previously described [12]. Fifty micrograms of proteins were loaded on 8% SDS-PAGE gel and transferred onto nitrocellulose membrane. The membrane was blocked with 5% (*w*/*v*) non-fat dry milk in phosphate buffer saline (PBS) with 0.1% (*v*/*v*) tween 20, before incubation overnight at 4 °C with anti-mouse CD73 polyclonal goat antibody 1:200 (Santa Cruz Biotechnology, Dallas, TX, USA). Then the membrane was incubated for 2 h at room temperature with the secondary antibody anti-goat IgG 1:2000 (Dako, CA, USA) conjugated with peroxidase. Anti-β-actin antibody (Santa Cruz Biotechnology) was used as a loading protein control. Protein bands were visualized using the ECL detection kit (Bio-Rad, Milan, Italy) and the ChemiDoc Imaging System (Bio-Rad, Italy). Densitometry was obtained using Image Lab software (Bio-Rad, Milan, Italy).

### 2.4. Measurement of Airway Responsiveness

From each animal group, the main bronchi were collected and rapidly cleaned from fat and connective tissue. Rings of 1–2 mm length were cut and mounted in 2.5 mL isolated organ baths containing Krebs solution, at 37 °C, oxygenated (95% O_2_ and 5% CO_2_), and connected to an isometric force transducer (type 7006, Ugo Basile, Comerio, Italy) associated to a Powerlab 800 (AD Instruments). Bronchial reactivity to cumulative concentrations of carbachol (10^−9^–3 × 10^−6^ M) was evaluated. Results were expressed as dyne per mg of tissue [15].

### 2.5. Measurement of AMPase Activity

AMPase activity was assessed in lung homogenates by the measurement of inorganic phosphate (Pi) released after incubation with AMP (2 mM) through a Malachite Green assay as previously described [12]. To confirm that Pi measured was dependent on CD73 enzymatic activity, each sample was also tested following incubation with the CD73 inhibitor, adenosine 5′- (α, β-methylene) diphosphate (APCP, 100 µM); the value of nonspecific Pi released was subtracted from the value obtained following incubation with AMP. Results were expressed as Pi released (pmol/min/µg protein).

### 2.6. Enzyme-Linked Immunosorbent Assay

Total IgE levels were quantified in plasma samples by ELISA kit (BD Pharmingen, Franklin Lakes, NJ, USA or R&D Systems, Inc., Minneapolis, MN, USA) according to the manufacturer’s instructions.

### 2.7. Lung Histology

Lung lobes were removed, fixed in 4% formalin, and embedded in paraffin. The tissue was then sectioned into 7 μm slices and stained with haematoxylin and eosin (H & E) for morphological analysis. In addition, to detect mast cells in the lung, tissue sections were stained with toluidine blue. Toluidine blue-positive mast cell numbers and degranulated mast cells were determined as previously described [16].

### 2.8. Flow Cytometry Analysis

Lungs were digested with 1 U/mL collagenase (Sigma-Aldrich, Milan, Italy). The analysis of inflammatory cells was determined by flow cytometry (BD FacsCalibur Milan, Italy) using the following antibodies: B220-Phycoerythrin (PE), CD19-Phycoerythrin-Cyanine5 (Pe-Cy5), CD23-Allophycocyanin (APC), CD3-Peridinin chlorophyll (PerCP), and CD4 Allophycocyanin-Cyanine7 (APC-Cy7). Intracellular staining for IL-4 (IL4-PE) was performed on fixed/permeabilized T cells after the extracellular staining for CD3 and CD4.

### 2.9. Statistical Analysis

All results are presented as mean ± standard error (SE). Statistical analysis was performed through Student’s *t*-test for unpaired data or by one- or two-ways ANOVA for multiple comparisons followed by Bonferroni post-hoc test, as appropriate. A *p* value < 0.05 was considered statistically significant. 

## 3. Results

### 3.1. Pulmonary Tissues from Ovalbumin-Challenged Mice Show High Levels of CD73 Expression and Activity

**Figure 1:**
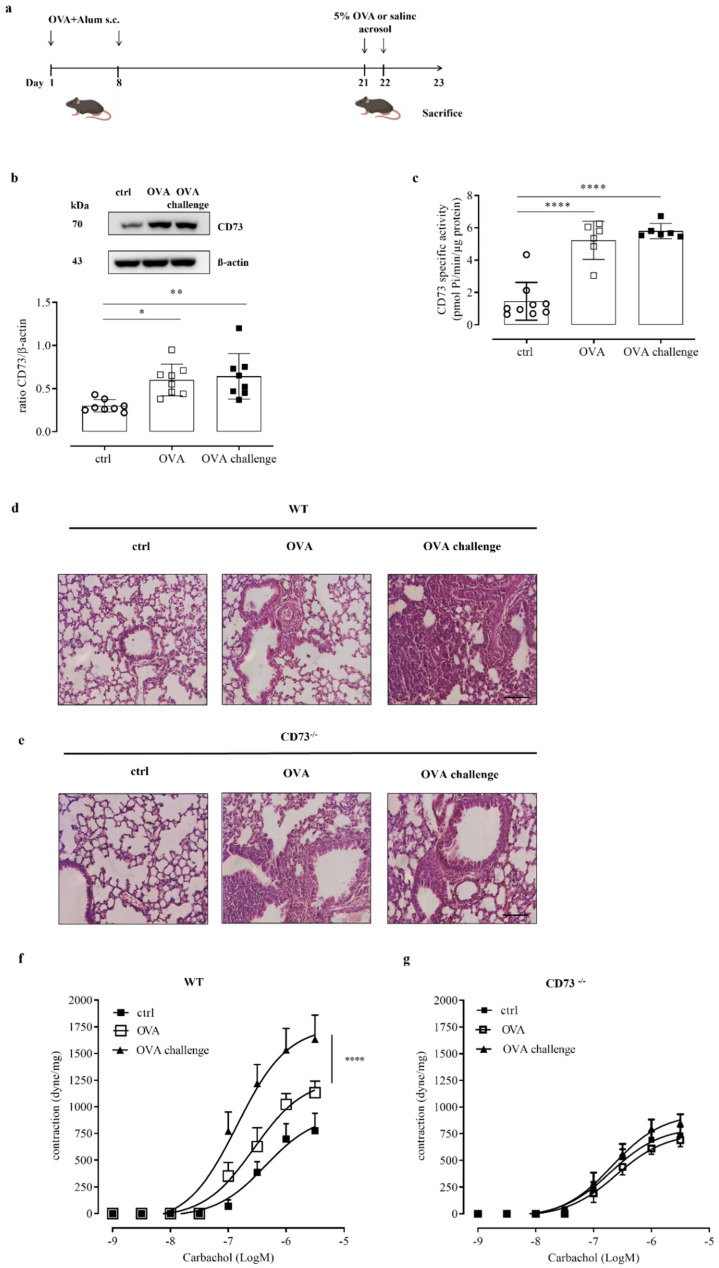
(**a**) OVA sensitization and aerosol challenge protocol in WT and CD73^−/−^ mice. Mice were sensitized and challenged with OVA as described in Materials and Methods. (**b**) CD73 pulmonary expression in control and OVA-sensitized and challenged WT mice was evaluated by Western blots. Data are expressed as means ± SE of eight mice per group. * *p* < 0.05 and ** *p* < 0.01 (one-way ANOVA followed by Bonferroni’s Multiple Comparison Test). (**c**) AMPase activity was determined by Malachite Green assay in lung tissue from control and OVA-sensitized and challenged WT mice. All data are expressed as mean ± SE of six to nine mice per group. **** *p* < 0.0001 (one-way ANOVA followed by Bonferroni’s Multiple Comparison Test). Histopathological changes in the lung tissues from control and sensitized and challenged WT (**d**) and CD73^−/−^ mice (**e**) were assessed by hematoxylin and eosin (H&E) staining. Scale bar: 100 µm. Bronchial reactivity in response to carbachol was evaluated in control and OVA-sensitized and challenged WT (**f**) and CD73^−/−^ (**g**) mice; * *p* < 0.05 and **** *p* < 0.0001; two-ways ANOVA plus Bonferroni.

In order to investigate the role of CD73 in controlling the airway response to an allergen challenge, we used an ovalbumin-sensitized/challenged mouse model (Figure 1a). Mice were subcutaneously sensitized with ovalbumin (OVA), at day 1 and 8, followed by aerosolized OVA-challenge (OVA-challenge) for 2 consecutive days (day 21 and day 22) to induce allergic airway inflammation (Figure 1a). Mice were sacrificed at day 23. We first analyzed the expression and activity of CD73 in whole-lung tissue samples. CD73 expression and AMPase activity were up-regulated in lung tissue samples following sensitization, without a further increase following OVA-challenge (Figure 1b,c, respectively). The AMPase activity was completely reversed in the presence of the selective inhibitor of CD73, APCP (data not shown).

### 3.2. Ovalbumin Challenge Does Not Increase Pulmonary Inflammatory Cell Infiltration in CD73^−/−^ Mice

When we examined pulmonary morphology and inflammation by hematoxylin and eosin staining, we observed that the recruitment of inflammatory cells in the peribronchial region was markedly increased following OVA-challenge compared with lung sections from OVA-sensitized mice and control mice (Figure 1d) in WT mice. We noticed that the recruitment of inflammatory cells in the peribronchial region of lung from CD73-deficient mice (CD73^−/−^) resulted as markedly increased after sensitization, as we have previously demonstrated [14], without a further increase following the OVA challenge (Figure 1e).

### 3.3. Ovalbumin Challenge Does Not Increase Bronchial Hyperreactivity to Carbachol in CD73^−/−^ Mice

We evaluated the role of CD73 in the assessment of bronchial hyperreactivity triggered by an allergen challenge. Bronchial reactivity was evaluated as a response to carbachol of OVA-sensitized and challenged WT and CD73^−/−^ mice. OVA sensitization increased bronchial reactivity to carbachol in WT mice, while it did not affect bronchial reactivity to carbachol in CD73^−/−^ mice [14]. Here, we found that OVA challenge significantly increased bronchial reactivity to carbachol of sensitized WT mice (Figure 1f) but not in sensitized CD73^−/−^ mice (Figure 1g).

### 3.4. Ovalbumin Challenge Enhances IgE Production in All Mice Groups

**Figure 2:**
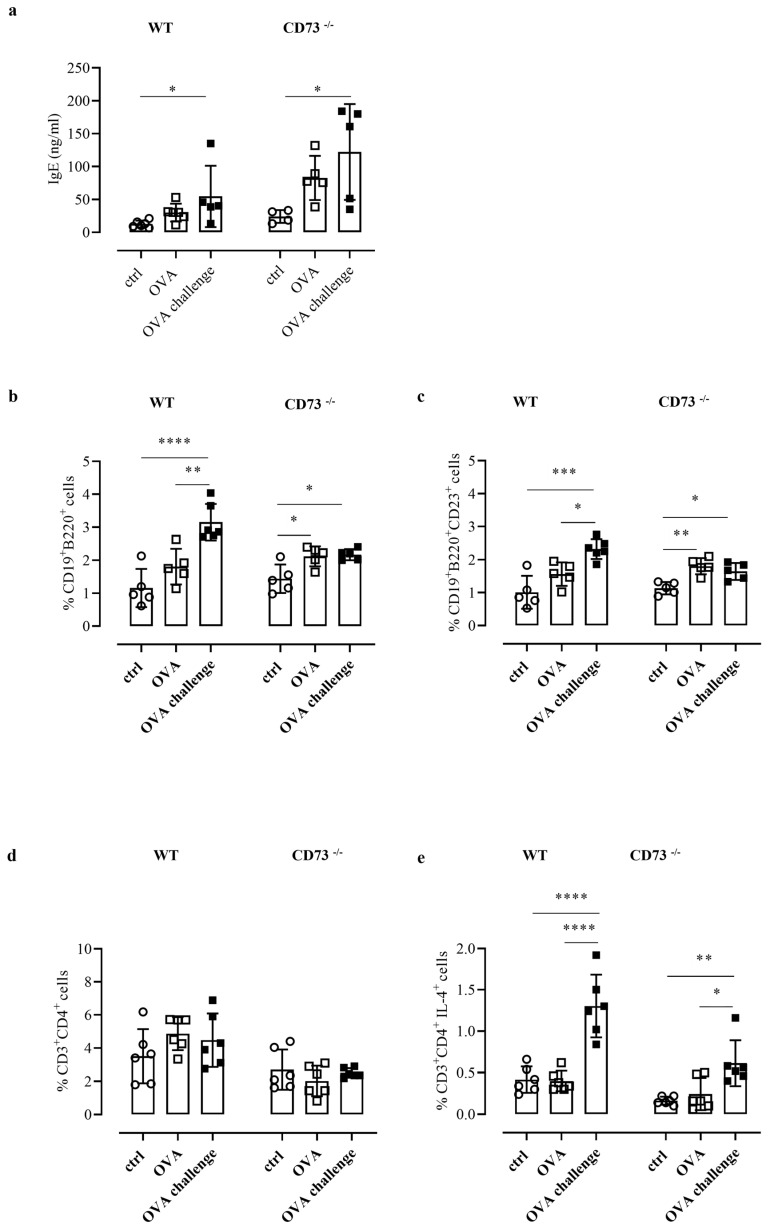
(**a**) Plasma levels of total IgE were measured in control and OVA-sensitized and challenged WT and CD73^−/−^ mice by ELISA. All results are expressed as mean ± SE of n = 4–6 mice per group. * *p* < 0.05 (one-way ANOVA followed by Bonferroni’s Multiple Comparison Test). The % of CD19^+^B220^+^ was evaluated in control and sensitized and challenged WT and CD73^−/−^ mice (**b**) by flow cytometry as well as the % of CD19^+^B220^+^CD23^+^ (**c**). Data are expressed as means ± SE of five to six mice per group. * *p* < 0.05, ** *p* < 0.01,*** *p* < 0.001, **** *p* < 0.0001 (one-way ANOVA followed by Bonferroni’s Multiple Comparison Test). The % of CD3^+^CD4^+^ and CD3^+^ CD4^+^ IL4^+^ in control and sensitized and challenged WT and CD73^−/−^ mice was analyzed by flow cytometry (**d**,**e**). Data are expressed as means ± SE of five to six mice per group. * *p* < 0.05, ** *p* < 0.01, **** *p* < 0.0001 (one-way ANOVA followed by Bonferroni’s Multiple Comparison Test).

Analysis of total IgE levels was performed after ovalbumin challenge in plasma of WT and CD73^−/−^ mice, as described in the Method section, and compared with plasma IgE levels determined in sensitized and control mice. The OVA challenge increased IgE levels in sensitized WT and CD73^−/−^ mice, compared with their respective non-sensitized controls; notably, IgE levels following OVA challenge were significantly higher in sensitized CD73^−/−^ mice than in their sensitized WT counterpart (122.2 ± 32.60 ng/mL, vs. 54.68 ± 20.86 ng/mL, n = 5; *p* < 0.05) (Figure 2a). Increased IgE levels in CD73^−/−^ mice compared with WT mice were also observed before OVA challenge (Figure 2a).

### 3.5. OVA Challenge Increases Pulmonary Infiltration of CD23^+^B Cells and IL-4^+^ T Cells

We then analyzed the percentage of B cells (as CD19^+^ B220^+^ cells) in pulmonary tissues from all groups of WT mice and CD73^−/−^ mice by flow cytometry. We found that OVA challenge increased the percentage of B cells in tissue samples from sensitized WT mice compared with control mice (Figure 2b). In tissue samples from sensitized CD73 ^−/−^ mice we found an increased frequency of B cells, compared to control mice, which were not further increased following OVA-challenge (Figure 2b). When we analyzed the expression of the low-affinity receptor for IgE (CD23) that is crucial in the IgE-induced inflammatory responses under allergen exposure [17], we observed that B cells were almost exclusively CD23^+^ (Figure 2d). Indeed, the percentage of CD23^+^B cells in WT mice following OVA challenge was significantly increased compared with sensitized WT mice (Figure 2c); conversely, in CD73^−/−^ mice following OVA challenge the percentage of CD23^+^B cells was increased compared with control mice but similar to that observed in sensitized CD73^−/−^ mice (Figure 2c). 

CD23 is crucial in the IgE-allergen-induced activation of T cells toward a Th2-like phenotype [17]. Th2-derived IL-4 represents an important effector mechanism of type 1 allergy [1]. Therefore, we next analyzed the percentage CD4^+^ T cells expressing IL-4 in pulmonary tissues from all groups of WT mice and CD73^−/−^ mice by flow cytometry analysis. Analyzing the total levels of CD3^+^CD4^+^T cells, we did not observe any significant changes among the experimental groups (Figure 2d). Instead, the frequency of CD3^+^CD4^+^IL-4^+^ cells was significantly increased in both WT and CD73^−/−^ OVA-challenged mice groups (Figure 2e). Of note, we also observed that the percentage of IL4^+^T cells in CD73^−/−^ mice, although they were significantly increased after OVA challenge, did not reach the frequency triggered by OVA challenge in WT mice (0.61 ± 0.11%, n = 6 vs. 1.30 ± 0.15%, n = 6; *p* < 0.0001 Bonferroni test) (Figure 2e). 

### 3.6. Ovalbumin Challenge Does Not Increase Mast Cell Degranulation in the Lung of CD73^−/−^ Mice

**Figure 3:**
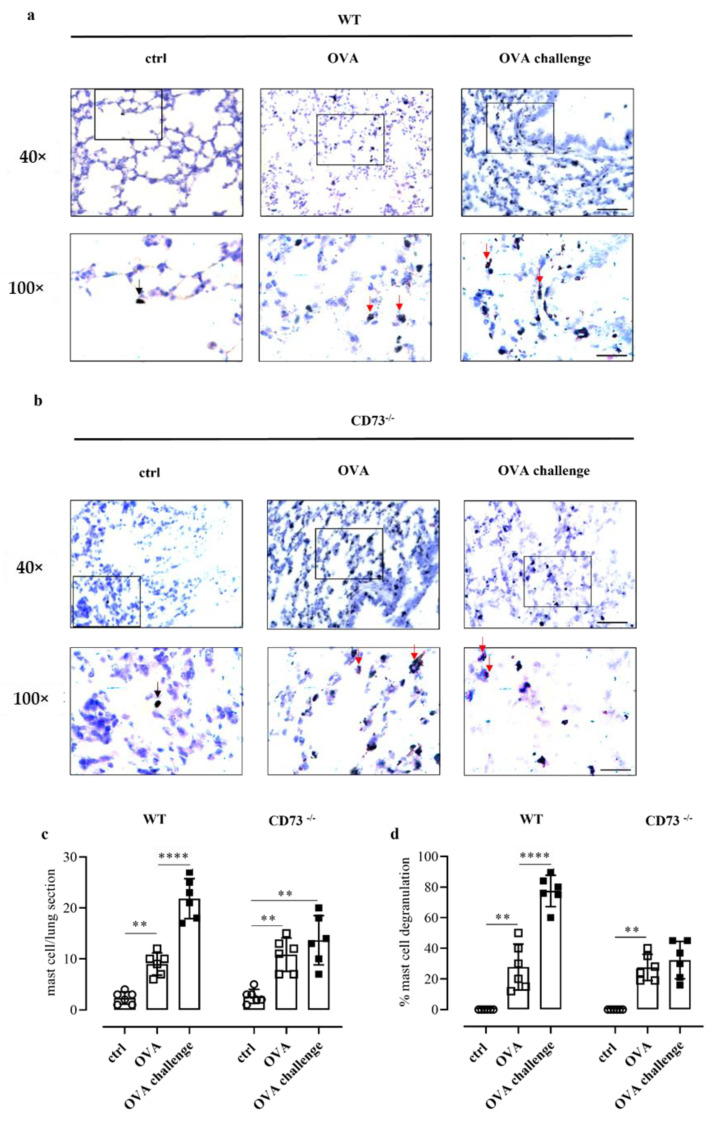
Representative photomicrographs of toluidine blue-stained lung sections from control and OVA-sensitized and challenged WT (**a**) and CD73^−/−^ mice (**b**). The number of pulmonary mast cells as well as a differentiation between non-degranulated (deep blue, black arrows) and degranulated (light blue, red arrows) and the percentage of mast cell degranulation was performed (**c**,**d**). Scale bar: 50 µm and 20 μm. Data are expressed as means ± SE of six mice per group. ** *p* < 0.01 and **** *p* < 0.0001 (one-way ANOVA followed by Bonferroni’s Multiple Comparison Test).

Pulmonary mast cell number and degranulation were then evaluated by means of toluidine blue staining in WT and CD73^−/−^ mice following sensitization and challenge. OVA sensitization increased the number of mast cells in the lung of WT (Figure 3a,c) and CD73^−/−^ mice (Figure 3b,c). However, OVA challenge further increased the number of pulmonary mast cells in sensitized WT mice, whereas it did not affect the number of pulmonary mast cells in sensitized CD73^−/−^ mice (Figure 3c). Concomitantly, OVA challenge increased the percentage (%) of degranulated mast cells in sensitized WT mice but not in CD73^−/−^ mice (Figure 3d). 

## 4. Discussion

We have previously shown that CD73 plays a role in the assessment of allergic sensitization, a requisite for an allergic reaction to occur. Indeed, in a model of OVA-sensitized CD73^−/−^ mice we observed exacerbation of airway inflammation that was paralleled by increased pulmonary inflammatory cytokine levels and a reduced percentage of pulmonary Foxp3^+^Tregs, suggesting that CD73 is functional to those surveillance mechanisms of the immune response [14]. 

Here, we investigated whether CD73 plays a role in the effector phase of allergic reaction. Thus, we developed the model of sensitized/allergen-challenged mice to compare functional and cellular response to the allergen challenge of sensitized WT and CD73^−/−^ mice.

According to our previous results, we found that mice sensitization with OVA increased airway CD73 expression and activity [14]; however, there was no further increase following mice challenge with aerosolized OVA. The functional study showed hyperreactivity to carbachol of bronchi from sensitized mice and a further increase of reactivity of bronchi from allergen-challenged sensitized mice. Likely, CD73 up-regulation, following sensitization, primes bronchi to ova challenge, while hyperreactivity to carbachol, which was observed in sensitized WT but not CD73^−/−^ mice, may be dependent upon adenosine availability into the airways [7].

Interestingly, in CD73^−/−^ mice, neither sensitization alone nor sensitization plus an allergen challenge caused bronchial hyperreactivity, suggesting that the lack of CD73, despite causing allergic airway inflammation, as previously demonstrated, gives protection from hyperreactivity caused by an allergen challenge. Schreiber et al. [18] described the absence of airway hyperresponsiveness to an allergen in CD73^−/−^ mice, of both sexes, due to inhibition of the tracheal Cl^−^ secretion stimulated by ATP and ADP following OVA challenge. 

Here, we investigated cellular mechanisms that are differently activated by an allergen challenge in sensitized WT and CD73^−/−^ mice. First of all, we found that in all groups of animals, circulating IgE levels were increased by an allergen challenge, indicating that the absence of CD73 did not affect the ability of B cells to produce IgE in response to an allergen challenge. This result suggests that the functional difference between sensitized WT and CD73^−/−^ mice, following an allergen challenge, probably lies in effector mechanisms triggered by an allergen challenge in WT and CD73^−/−^ mice. 

It is worth noting that IgE levels from CD73^−/−^ were higher than levels obtained from WT mice. The significance of such a difference needs to be further investigated; however, it was demonstrated that the couple CD39/CD73, through increasing adenosine levels, plays a role in Ig class switch in B cells [19]; on this basis, we could hypothesize that, under our experimental conditions, Ig class switch would be lost in B cells from CD73^−/−^ mice, resulting in high IgE levels. 

Besides the high affinity IgE receptor, FcεRI, on effector cells of type I allergy, another receptor for IgE, CD23 (FcεRII), represents an important modulator of immune response since it regulates IgE levels. CD23 on B cells is a low-affinity receptor for IgE, however, it can oligomerize, leading to enhanced IgE binding [17]. It is known that when allergen crosslinks the IgE-FcεRI receptor, effector cells are activated and an allergic response occurs. Evidence that there is a reciprocal inhibition between FcεRI and CD23 that blocks simultaneous binding by IgE suggested that these two receptors have different roles in the control of IgE [20,21]. We found that the OVA challenge increased the percentage of CD23^+^ B cells into the pulmonary tissue of sensitized WT mice but not in sensitized CD73^−/−^ mice. 

It is known that the density of CD23 molecules on B cells correlates with IgE levels and facilitates allergen-specific T-cell activation [22]. Thus, there is a link between the Th2 response and CD23 density; in agreement, it has been shown that an anti-CD23 antibody inhibits Th2 response [23]. Increased levels of CD23^+^B cells in patients with grass pollen allergy have been shown [22]. Thus, under our experimental conditions, we observed a reduced frequency of pulmonary CD23^+^B cells in sensitized/OVA-challenged CD73^−/−^ mice associated to a protection from allergen-induced airway hyperreactivity. It is worth noting that the difference in the frequency of pulmonary CD23^+^B cells between WT and CD73^−/−^ mice was observed following an OVA challenge but not before, in only sensitized mice, meaning that in CD73^−/−^ mice, B cell response to an allergen challenge is defective, which is in agreement with an important role of CD73 in memory B cells development [24,25].

On this basis, we evaluated the frequency of pulmonary IL-4^+^Tcells in our experimental groups, with or without an allergen challenge. We found that an allergen challenge increased the frequency of CD3^+^CD4^+^IL-4^+^ cells in airways in both groups of OVA-sensitized WT and CD73^−/−^ mice, with an increase in WT mice significantly higher than that achieved in CD73^−/−^ mice. This result suggests that the lack of CD73 reduces Th2 response following an allergen challenge.

Finally, we also found that OVA challenge increased pulmonary mast cell accumulation and degranulation only in the WT-sensitized group but not in CD73^−/−^-sensitized groups. This result requires further investigation to understand whether this is a key point. Mast cells represent effector cells of type I allergy, and their degranulation is mediated by allergens linking FcεRI-bound IgE and gives immediate allergic symptoms. The role of CD73/adenosine signalling in antigen-induced mast cell degranulation is still poorly understood; however, there is evidence that adenosine potentiates antigen-induced pulmonary mast cell activation [26].

In conclusion, our results demonstrate increased CD73 expression and activity in sensitized airways and the lack of airway response to an allergen challenge of CD73^−/−^-sensitized mice characterized by reduced Th2 activation and reduced mast cell accumulation and degranulation. Although further investigation is needed, our results provide the basis to reflect on CD73 as a potential therapeutic target for type I allergy. 

## Data Availability

The data that support the findings of this study are contained in the body of the manuscript. Any additional data used are available from the corresponding authors upon reasonable request.
